# Beyond Behavioral Inhibition: A Computer Avatar Task Designed to Assess Behavioral Inhibition Extends to Harm Avoidance

**DOI:** 10.3389/fpsyg.2017.01560

**Published:** 2017-09-15

**Authors:** Michael Todd Allen, Molly M. Jameson, Catherine E. Myers

**Affiliations:** ^1^School of Psychological Sciences, University of Northern Colorado, Greeley CO, United States; ^2^Department of Veterans Affairs, VA New Jersey Health Care System, East Orange NJ, United States; ^3^Department of Pharmacology, Physiology and Neuroscience, New Jersey Medical School, Rutgers University, Newark NJ, United States

**Keywords:** anxiety, avoidance, behavioral inhibition, personality assessment, virtual reality

## Abstract

Personality factors such as behavioral inhibition (BI), a temperamental tendency for avoidance in the face of unfamiliar situations, have been identified as risk factors for anxiety disorders. Personality factors are generally identified through self-report inventories. However, this tendency to avoid may affect the accuracy of these self-report inventories. Previously, a computer based task was developed in which the participant guides an on-screen “avatar” through a series of onscreen events; performance on the task could accurately predict participants’ BI, measured by a standard paper and pencil questionnaire (Adult Measure of Behavioral Inhibition, or AMBI). Here, we sought to replicate this finding as well as compare performance on the avatar task to another measure related to BI, the harm avoidance (HA) scale of the Tridimensional Personality Questionnaire (TPQ). The TPQ includes HA scales as well as scales assessing reward dependence (RD), novelty seeking (NS) and persistence. One hundred and one undergraduates voluntarily completed the avatar task and the paper and pencil inventories in a counter-balanced order. Scores on the avatar task were strongly correlated with BI assessed via the AMBI questionnaire, which replicates prior findings. Females exhibited higher HA scores than males, but did not differ on scores on the avatar task. There was a strong positive relationship between scores on the avatar task and HA scores. One aspect of HA, fear of uncertainty was found to moderately mediate the relationship between AMBI scores and avatar scores. NS had a strong negative relationship with scores on the avatar task, but there was no significant relationship between RD and scores on the avatar task. These findings indicate the effectiveness of the avatar task as a behavioral alternative to self-report measures to assess avoidance. In addition, the use of computer based behavioral tasks are a viable alternative to paper and pencil self-report inventories, particularly when assessing anxiety and avoidance.

## Introduction

Personality traits as well as behavioral tendencies play a role in vulnerability to psychiatric disorders including anxiety disorders. For example, behavioral inhibition (BI) is defined as a tendency to avoid or withdraw from unfamiliar people and situations (Kagan et al., 1987; Morgan, 2006). In childhood, BI is identified through structured interviews and /or observation of a child’s behavior when confronted with unfamiliar people and objects (Kagan et al., 1984). While childhood BI increases the risk for an individual to develop anxiety disorders in adulthood (Hirshfeld et al., 1992; Biederman et al., 1993; Svihra and Katzman, 2004; Pérez-Edgar et al., 2010), including post-traumatic stress disorder (PTSD; North et al., 1999; Fincham et al., 2008; Kashdan et al., 2009), not all behaviorally inhibited children will continue to exhibit these inhibited tendencies in adolescence and adulthood (Degnan and Fox, 2007). We have investigated a learning diathesis model of anxiety disorders in which personality factors such as BI are associated with enhanced learning (Myers et al., 2012a,b; Sheynin et al., 2013; Allen et al., 2014, 2016; Holloway et al., 2014; Allen and Miller, 2016), a cognitive bias that might confer vulnerability for anxiety through enhanced association of previously neutral cues with the threat of aversive outcomes.

One issue is how BI is measured. BI is identified in childhood based on structured interviews and/or observations of behavior (Kagan et al., 1984). In adulthood, BI is most often assessed through paper and pencil self-report inventories, which ask respondents to rate themselves relative to implicit social norms. These inventories include the Retrospective and Concurrent Self-Report of Inhibition (RCSRI; Reznick et al., 1992) and the BIS/BAS Scale (Carver and White, 1994). While the RCSRI works well with childhood BI, it has not been studied much with adult populations. The BIS scale is correlated with trait anxiety and harm avoidance (HA; Carver and White, 1994) and has been found to be internally consistent (Jorm et al., 1998). However, the BIS/BAS self-reported measures have been found to be relatively independent of psychophysiological measures (Brenner et al., 2005). Therefore, this inventory deals more with feelings about consequences of BIS/BAS than the behaviors themselves (Carver and White, 1994; Leone et al., 2001). There are also issues in that these inventories ask the respondents to self-evaluate their behaviors compared to perceived social norms; subjects’ self-report may suffer from a lack of awareness of their feelings or social norms.

Rather than have respondents report on their feelings as compared to hypothetical social norms, the Adult and Retrospective Measures of Behavioral Inhibition (AMBI/RMBI; Gladstone and Parker, 2005; Gladstone et al., 2005) asks respondents to report on specific behaviors such as “Are you likely to spend most of your time next to a person you know well?” AMBI scores not only predict anxiety vulnerability (Gladstone et al., 2005) but also correlate with PTSD symptoms (Myers et al., 2012a,b). Recent work with undergraduates (Allen et al., 2014, 2016; Holloway et al., 2014), as well as veterans with severe PTSD symptoms (Myers et al., 2012a,b), has utilized the AMBI to identify anxiety vulnerable individuals who exhibit enhanced associative learning. The AMBI and the other above mentioned questionnaires have thus proven useful in elucidating the construct of BI and its relation to risk of anxiety and PTSD.

However, there are inherent limitations to the use of any self-report questionnaire. The most obvious limitation is the potential for response bias and demand characteristics (see McCambridge et al., 2012). A more specific problem with self-reports of BI involves the tendency for behaviorally inhibited individuals to avoid. Avoidance is the prototypical behavior of many anxiety disorders including acute stress disorder, separation anxiety disorder, and PTSD (American Psychiatric Association, 2013). Avoidant behaviors have been hypothesized to distinguish between those at risk for an anxiety disorder and those who are not (North et al., 1999; Barlow, 2002; Karamustafalioglu et al., 2006; Marshall et al., 2006; O’Donnell et al., 2007). However, a tendency to avoid may affect the accuracy of self-report both in non-clinical and clinical settings. Undergraduates may seek to look good in the eyes of the researcher to avoid drawing attention to their actual tendency for avoidance. Participants within a clinical setting may exaggerate their responses toward non-avoidance related choices to avoid the diagnosis of PTSD. Professionals, such as emergency workers, who develop PTSD while on the job may avoid participation in research with PTSD questionnaires due to fear of losing their jobs (Clohessy and Ehlers, 1999).

For these reasons, an objective behavioral measure of BI was developed as a computer-based task in an interactive virtual environment by Myers et al. (2016a). In the task, participants select an avatar to represent them in a series of social scenarios in which they choose how they would respond in real life. Myers et al. (2016a) demonstrated that scores on the avatar task could accurately predict participants’ scores on the AMBI. In subsequent work, Myers et al. (2016b) replicated the strong relationship between scores on the avatar task and the AMBI in a sample of veterans, and also reported that scores on the avatar task positively correlated with PTSD symptom severity.

The first aim of the present work was simply to replicate and extend the prior findings, by testing the generalizability of the avatar task to a sample from a different racial/ethnic population in a different region of the country. Specifically, Myers et al. (2016a) reported findings from a mainly female sample drawn from undergraduate students at a small urban campus in the Northeast with a large minority population. In their follow-up study with veterans with PTSD, Myers et al. (2016b) reported findings from an older, largely minority sample of mainly male Veterans in the Northeast. It is possible that the fairly high rates of BI observed in these prior samples reflect an urban orientation: for example, AMBI items such as, “Do you tend to become vigilant and wary of your surroundings?” or “Do you tend to keep a fair distance away from strangers?” might be more likely to trigger endorsement in city dwellers, inflating group BI scores due to environment rather than personality. Therefore, to examine the generality of the relationship between BI and avatar task, we administered both the avatar task and AMBI to a new non-clinical sample, consisting of college students recruited from a Western university in a smaller mixed urban and rural environment with a mainly Caucasian population.

A second aim of the present work was to evaluate whether avatar scores also correlated with anxiety vulnerability, as assessed by other self-report measures. The avatar task scenarios and items were developed based on the types of behaviors assessed in AMBI, and thus it would be expected that the task produces scores that correlate with AMBI scores. In the current work, we sought to extend these findings by examining the relationship between avatar scores and a different temperamental measure related to anxiety vulnerability, HA. HA is defined as a tendency to respond strongly to aversive stimuli and learn to avoid punishment, novelty, and non-reward (Nixon and Parsons, 1989). HA also includes excessive worrying; pessimism; shyness; and being fearful, doubtful, and easily fatigued (Cloninger, 1986, 1987). HA is measured with a scale of the Tridimensional Personality Questionnaire (TPQ; Cloninger et al., 1991, 1993). HA consists of four subscales which address anticipatory worrying, fear of uncertainty, shyness, and fatigability. The anticipatory worry (HA1) subscale measures pessimism and anticipating worry compared to uninhibited optimism. The fear of uncertainty (HA2) subscale measures tension about uncertainty or physical danger as compared to confidence. The shyness (HA3) subscale measures shyness with strangers as compared to gregariousness. The fatigability (HA4) subscale measures fatigability or asthenia as compared to vigor.

Finally, the third aim of the present work was to examine the specificity of the relationships between task performance and anxiety vulnerability. For example, rather than a selective relationship with the psychological construct of BI, it might be that subjects who score highly on the avatar task might simply be prone to choose more “extreme” questionnaire responses. An advantage of the TPQ over unidimensional questionnaires such as AMBI is that the TPQ also includes three other subscales in addition to HA: novelty seeking (NS), reward dependence (RD) and persistence (Cloninger et al., 1993). NS behaviors include frequent exploratory activity and heightened responses to novel or appetitive stimuli (Wills et al., 1994), while RD includes a marked response to rewarding stimuli and a resistance to extinction. Persistence was originally a subscale of RD, but was separated from RD in a subsequent revision of the TPQ (Cloninger et al., 1993); persistence measures behaviors including perseverance despite frustration and fatigue (Maremmani et al., 2005). These subscales of the TPQ offer the opportunity to test for the ability of the avatar task to differentiate avoidant and non-avoidant behaviors. It would expected that HA would be strongly correlated with behaviors on the avatar task, but that NS, RD, and Persistence would not, confirming a selective association of avatar task with avoidant temperament. We also sought to examine whether BI and HA (and the HA subscales) interact to predict scores on the avatar task.

In the current study, we hypothesized a strong positive relationship between scores on the avatar task and BI and HA, while expecting no positive relationships with other personality factors not related to avoidance and BI (i.e., NS, RD, persistence). We also hypothesized that the standard cut-off scores for the AMBI and HA, defining “high-BI” or “highly harm-avoidant” individuals, would be able to differentiate avatar scores.

## Materials and Methods

### Participants

One hundred and one undergraduate students enrolled in a large Western University, including both rural and urban components, voluntarily completed this study for partial research credit for an introductory psychology course. Our sample included 51 females and 50 males with a mean age of 18.7 years (*SD* = 2.7, range 18–44 years) and a mean education level of 12.5 years (*SD* = 0.93). A majority of subjects self-reported race/ethnicity as Caucasian (*n* = 69), followed by Hispanic (*n* = 17), African–American (*n* = 6), East Asian (*n* = 3), South Asian, (*n* = 1), multi-racial (*n* = 1), and other (*n* = 4).

### Instruments

Participants completed a short questionnaire about their demographic information including gender, age, years of education, and race/ethnicity. Participants then completed the paper and pencil Adult Measure of Behavioral Inhibition or AMBI questionnaire (Gladstone and Parker, 2005). The questionnaire contains 16 questions in which the participant reports on current (adult) behavior when entering a new or unfamiliar social situation, or new and unfamiliar surroundings. For each item, the respondent indicates from three responses options whether the behavior described for each item is true for them “most of the time,” “some of the time,” or “hardly ever.” Possible AMBI scores range from 0 to 32, with higher scores indicating higher levels of BI. Previously published cut-points suggest classification of individuals scoring 15.5 or higher as behaviorally inhibited or “BI” and the remainder as non-inhibited or “NI” (Gladstone and Parker, 2005).

Participants also completed the Tridimensional Personality Questionnaire (TPQ; Cloninger et al., 1991). This self-report questionnaire consists of 100 true/false items assessing how the individual feels or behaves in various daily situations, and provides scores relating to three orthogonal personality dimensions, HA, NS, and RD, which was later subdivided into RD and Persistence. HA consists of 33 items, and is defined as personality related to BI in response to novel or aversive situations (Cloninger, 1986, 1987). Based on previously published cut-points, individuals scoring 12 or higher on HA are classed as highly harm avoidant or “HA” and the remainder as non-avoidant or “non-HA” (Cloninger et al., 1991).

Harm avoidance consists of four subscales, anticipatory worry, fear of uncertainty, shyness, and fatigability. The anticipatory worry subscale includes 11 items such as “I am usually confident that everything will go well, even in situations that worry most people.” The fear of uncertainty subscale includes 7 items such as “I usually feel tense and worried when I have to do something new and unfamiliar.” The shyness subscale includes 7 items such as “I often avoid meeting strangers because I lack confidence with people I do not know.” The fatigability subscale includes 10 items such as “I try to do as little work as possible even when other people expect more of me.”

The other two dimensions originally assessed by the TPQ are RD and NS. The RD scale consists of 33 items includes items such as “I usually push myself harder than most people do because I want to do as well as I possibly can.” The NS scale consists of 34 items and includes items such as “I often try new things just for fun or thrills, even if most people think it is a waste of time.” The persistence scale consists of nine items and includes items such as “I often push myself to the point of exhaustion or try to do more than I really can.”

### Computer-Based Task

**FIGURE 1:**
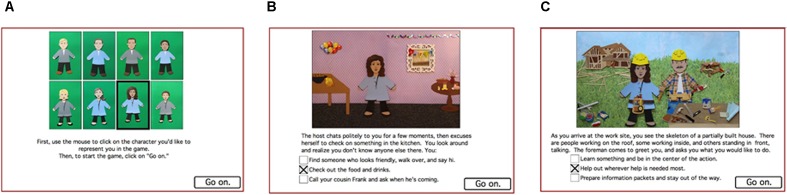
Sample screenshots from the computer-based task (Myers et al., 2016a). **(A)** Participants selected a character (“avatar”) to represent them in the task; here the female in the lower row is selected. **(B)** In the first scenario, the avatar is invited to a party where s/he does not know any of the people present except his/her cousin; here the avatar has the choice to interact with someone they don’t know. **(C)** In the second scenario, the avatar participates in a volunteer construction activity in which the avatar has to interact with other volunteer workers. In both scenarios, at each choice point, response options include relatively behaviorally inhibited, relatively non-inhibited, or intermediate behaviors.

The computer-based task was as previously described (Myers et al., 2016a); an open-access version of the task software is available at Open Science Framework www.osf.io/zf3jv. The task took about 10 min for participants to complete. First, participants were presented a screen with a selection of male and female avatars (**Figure 1A**), and were asked to choose one to represent them in the task. The task itself consisted of two scenarios, which involved attending a party full of strangers and volunteering to help on a charity building project. The script included 20 decision points; each decision point included a short text description and an image showing the avatar experiencing an event. The participant was presented with three response options including relatively avoidant, relatively non-avoidant, and neutral actions (**Figures 1B,C**). The script was designed so that the same sequence of events and response options appeared for all participants, regardless of their responses, although this lack of contingency should not have been obvious to the participant. At each choice point, the participant received two points for selecting the avoidant action, one for the intermediate action, and zero for the non-avoidant action. Total scores could range from 0 (least avoidant) to 40 (most avoidant). Scores were not displayed to the participant.

At the completion of the computer based task, participants completed a five question post task questionnaire (Myers et al., 2016a), asking about familiarity with computers, computer game play, whether they had been able to find an avatar they liked to represent them and whether they cared what happened to the avatar in the game, and the degree to which participants had made decisions in the game that were similar to how they act in real life.

### Data Analysis

Gender effects were analyzed with an independent-measures *t*-test. Pearson’s product moment correlation was calculated between total scores on the avatar task and the scores from the TPQ, with a Bonferonni correction for repeated correlations which resulted in the significance threshold being reduced from 0.05 to 0.005. Chi square analysis of gender effects were done on under-reporting and over-reporting in the AMBI and HA scales as compared to performance on the avatar task. We also utilized a multiple regression analysis and an indirect effects analysis to explore the relationship between scores on the avatar task and AMBI and HA scores. Finally, a univariate ANOVA was used to test whether the standard cut-off scores for BI and HA would result in significant differences in avatar scores.

## Results

Out of our total sample of 101 participants, a total of 93 participants consisting of 49 females and 44 males completed the avatar task and all items on both the AMBI and TPQ. All analyses are based on these 93 individuals.

### Paper and Pencil Inventories

Mean scores for the AMBI, HA scale of the TPQ, the four HA subscales, and the three other scales of the TPQ for all participants, and separately for females and males, are shown in **Table 1**. Females had higher HA scores than males, both for the overall HA score and also for the anticipatory worry, fear of uncertainty, and fatigability HA subscales. There were no significant gender differences in scores on the AMBI or the remaining TPQ subscales.

**Table 1:** Mean avatar score and inventory scores and gender effects.

Measures	Total mean (*SD*)	Female mean (*SD*)	Male mean (*SD*)	Gender effect significance level
Avatar score	17.4 (5.3)	17.8 (5.6)	17.1 (3.1)	ns
AMBI	16.0 (5.1)	16.7 (4.8)	15.2 (5.4)	ns
Harm avoidance	12.4 (7.3)	14.6 (7.1)	10.0 (7.2)	0.001
Anticipatory Worry	3.3 (2.5)	3.8 (2.2)	2.5 (2.6)	0.001
Fear of Uncertainty	3.0 (2.0)	3.5 (2.3)	2.3 (1.7)	0.001
Shyness	2.8 (2.1)	2.9 (2.0)	2.5 (2.1)	ns
Fatigability	3.3 (2.7)	4.2 (3.2)	2.4 (2.0)	0.001
Novelty seeking	16.2 (5.6)	16.8 (6.2)	15.6 (5.1)	ns
Reward dependency	19.1 (4.0)	19.9 (4.2)	18.2 (5.1)	ns
Persistence	6.1 (2.2)	6.3 (2.0)	6.0 (2.3)	ns

Based on the finding that females in our sample exhibited higher HA and HA subscales scores than males, we corrected for this gender difference in subsequent analyses with gender- corrected HA scores. Specifically, we performed a linear regression model gender (male = 0, female = 1) and HA as possible predictors of avatar scores, to generate weights (*B* value) for each predictor. The *B* value of gender (0.8) which corresponds to higher HA in females based on how the genders were coded in regression) was then subtracted from each raw HA score to calculate a gender-corrected HA score which was used in subsequent analyses.

On the post-test questionnaire, all but eight participants reported moderate or high familiarity with computers (*M* = 3.3, *SD* = 0.6); 62 participants (66.7%) reported regularly playing computer games. There were no significant gender differences in computer familiarity or self-reported game-playing (*t*-tests, all *p*’s > 0.100), or types of games played (chi-square test, *p* > 0.100).

### Avatar Selection and Comparison to Real Life Behavior

All participants selected same-gender avatars, even though they had not been instructed to do so. In general, those participants self-reporting race/ethnicity as African-American or Hispanic selected darker skinned avatars while those self-reporting as Caucasian or Asian selected fairer skinned avatars. Almost all (94%) of participants reported that they had been able to find an avatar they liked. Those few subjects who were not able to find an avatar they liked reported the selection was “boring,” “too generic,” “too old,” or “not relevant” to themselves. Participants indicated moderate level of concern about what happened to the avatar (*M* = 2.8, *SD* = 0.8). Subjects also indicated a high correspondence between their responses on the avatar task and how they normally act in real life (*M* = 3.6, *SD* = 0.6).

### Responses on the Computer-Based Task

The mean total score on the avatar task was 17.4 (*SD* = 5.3). There was no significant difference in total avatar scores (*p* = 0.45) between males and females.

The relationships between the scores on the avatar task and the inventory scores are shown in a correlation matrix in **Table 2**. HA and BI scores were significantly correlated, which would be expected based on the overlap in avoidant behaviors that each construct includes.

**Table 2:** Summary of correlations of scores on the avatar computer task, AMBI, and TPQ scales and HA subscales.

Measure	1	2	3	4	5	6	7	8	9	10
(1) Avatar computer task	–									
(2) Behavioral inhibition	**0.64^∗^**	–								
(3) Harm avoidance	**0.46^∗^**	**0.47^∗^**	–							
(4) Anticipatory worry	**0.33^∗^**	**0.32^∗^**	**0.67^∗^**	–						
(5) Fear of uncertainty	**0.42^∗^**	**0.52^∗^**	**0.59^∗^**	**0.51**^∗^	–					
(6) Shyness	**0.44^∗^**	**0.65^∗^**	**0.50^∗^**	**0.36^∗^**	**0.38^∗^**	–				
(7) Fatigability	0.23	0.26	**0.67^∗^**	**0.51**^∗^	**0.56^∗^**	0.27	–			
(8) Novelty seeking	-**0.39^∗^**	-**0.36^∗^**	-0.21	-0.06	-0.28	-0.22	-0.03	–		
(9) Reward dependency	-0.20	**-0.37^∗^**	-0.06	0.11	0.01	-0.28	0.02	0.10	–	
(10) Persistence	-0.02	-0.03	-0.09	-0.09	-0.07	-0.15	-0.07	-0.19	0.10	–

^∗^*p < 0.005 based on Bonferroni correction for multiple correlations. Results for the HA scale and HA subscales are based on gender-corrected scores on these scales*.

**FIGURE 2:**
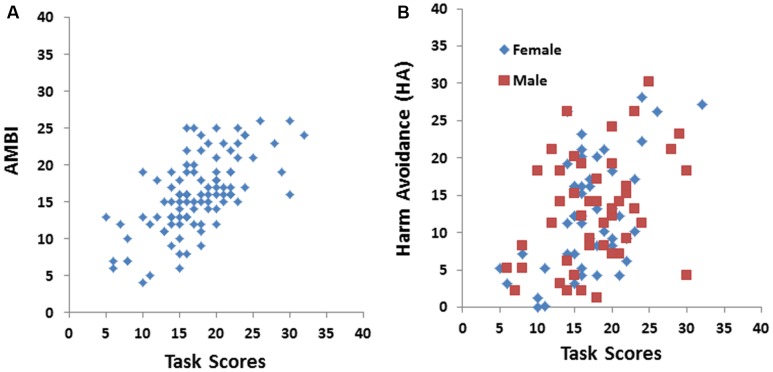
Total scores on the avatar task were strongly positively correlated with both **(A)** AMBI scores (*r* = 0.64) and **(B)** harm avoidance (HA) scores (*r* = 0.42).

The total score on the avatar task was strongly positively correlated with the AMBI score (*r* = 0.64, *p* < 0.001) as shown in **Figure 2A**, which replicated prior findings (Myers et al., 2016a,b). In addition, total score on the avatar task was also strongly positively correlated with the HA scale (*r* = 0.46, *p* < 0.001) as shown in **Figure 2B**. Females (*r* = 0.51) exhibited a stronger relationship between score the avatar task and the HA scale than males (*r* = 0.34).

**FIGURE 3:**
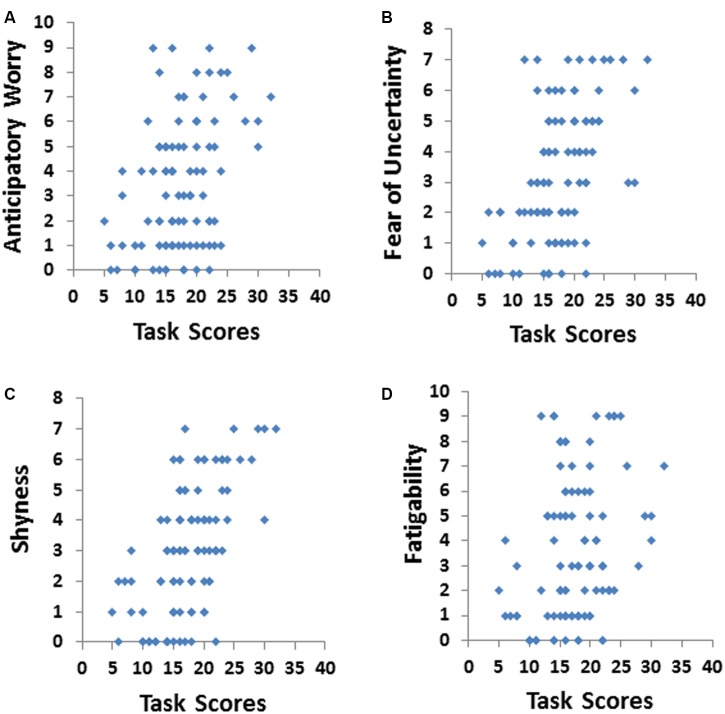
Total scores on the avatar task were strongly positively correlated **(A)** anticipatory worry (*r* = 0.33), **(B)** fear of uncertainty (*r* = 0.42), **(C)** shyness (*r* = 0.44), but not **(D)** fatigability (*r* = 0.23).

The HA scale of the TPQ consists of four subscales: shyness, fear of uncertainty, anticipatory worry, and fatigability. All of these HA subscales, except fatigability were significantly positively correlated with the total avatar score as shown in **Figure 3**. Shyness and fear of uncertainty had the strongest relationship to avatar scores, followed by anticipatory worry.

One possible explanation for this gender difference in the relationship between HA and avatar scores may be due to individuals whose self-reported inventory scores did not correspond with their performance on the avatar task. Specifically of interest are those who reported little tendency for BI or HA behaviors on the questionnaires but received high scores on the avatar task (i.e., subjects who may under-report avoidance compared to what they actually exhibit) and those who reported strong tendency for BI or HA behaviors on the questionnaires but received low scores on the avatar task (i.e., subjects who under-report avoidance compared to what they actually exhibit). Of particular interest was whether the same individuals who under-reported or over-reported did so both on the BI and HA scales. Of the 23 individuals who scored below the mean on BI (potentially under-reporting BI) and the 20 individual who scored below the mean HA (potentially under-reporting HA), there were only seven individuals who did so on both self-report inventories. In the case of over-reporting, a total of 17 individuals scored above the mean on HA (potentially over-reporting HA), with only four individuals scoring above the mean on BI (potentially over-reporting BI), although all four of these individuals also over-reported HA. Therefore, under-reporting or over-reporting was fairly independent between the BI and HA inventories. Given this finding, one could ask whether combining HA and BI produces a more accurate prediction of avatar scores than either measure alone. A multiple regression analysis revealed that gender and HA significantly predict avatar scores (*r* = 0.266, *p* = 0.037), but even though adding in AMBI increased the prediction (*r* = 0.286, *p* = 0.0054), this was not a significant improvement over gender and HA alone (chi-square of change, *p* = 0.307).

To further explore the impact of HA on the relationship between the avatar task and AMBI scores, an indirect effects analysis using Hayes (2013) PROCESS Macro for SPSS was conducted. With 1000 bootstrapped samples, this bias-corrected bootstrapped indirect effects analysis revealed a significant, yet small, indirect effect of HA on the relationship, *b* = 0.128, *bias-corrected (BCa) CI* [0.027,0.258]. This suggests that HA, or some component of HA, is mediating this existing relationship. To further explore this, the component scores were entered as mediating variables in the relationship between the avatar task and AMBI scores. **Table 3** shows the results for all component scores, with only fear of uncertainty showing an indirect effect, *b* = 0.105, *BCa CI* [0.030,0.215], suggesting that fear of uncertainty is a moderate mediating variable in this relationship. To explore whether this effect was simply the result of intercorrelations among study variables, an indirect effects analysis was conducted to determine if AMBI scores had an indirect effect on the relationship between the avatar task and HA; this analysis failed to support the effect of AMBI scores on this relationship. This suggests the robustness of the finding that HA is serving as an indirect effect on the relationship.

**Table 3:** Indirect effects analysis of HA subscales on the relationship between AMBI scores and the avatar task.

Variable	Indirect effect	BCa lower CI	BCA upper CI
Anticipatory worry	0.002	-0.041	0.051
Fear of uncertainty	0.105^∗^	0.03	0.215
Shyness	0.052	-0.058	0.178
Fatigability	-0.023	-0.097	0.002

^∗^*Significant indirect effect*.

*BCa Lower CI, Bias corrected lower level confidence interval; BCa upper CI, Bias corrected upper level confidence interval*.

In addition, we were also interested as to whether gender played a role in under- or over-reporting on the inventories as compared to performance on the avatar task. To address the possibility, we utilized a chi square test of goodness of fit to analyze the proportions of males and females who over-reported in that their inventory scores were in the upper half of the range for BI or HA and their task scores in the lower half of the range for the avatar task. We repeated the same analysis for the four HA subscales as well as the NS, RD, and persistence scales. As shown in **Table 3**, there were no significant gender effects for those individuals over-reporting on inventories as compared to behavior on the avatar task.

We also analyzed the ratio of males and females who under-reported on inventories in that their inventory scores were in the top half of the possible inventory scores and in the lower half of possible avatar scores. Males were more likely than females to under-report fatigability (chi square = 4.00, *p* < 0.05), but there were no other significant gender effects for the other scales or subscales.

**FIGURE 4:**
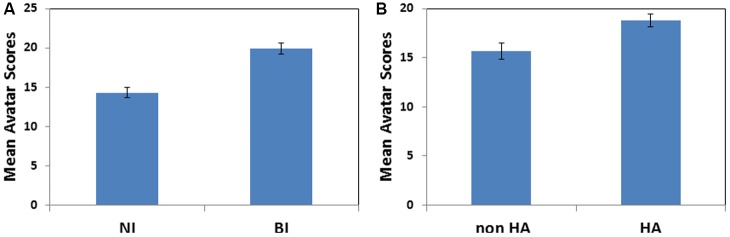
The standard cut-off scores for BI and HA scores can be used to differentiate total avatar scores. **(A)** BI individuals exhibited higher avatar scores than non-BI individuals. **(B)** HA individuals exhibited higher avatar scores than non-HA individuals.

To further explore the relationship between avatar scores and the inventory measures, we analyzed avatar scores in individuals classified as inhibited (BI) or non-inhibited (NI) based on AMBI, or as harm-avoidant (HA) or non-avoidant (non-HA) based on TPQ. BI individuals (*n* = 49, mean = 19.5, *SD* = 3.3) exhibited higher avatar scores than non-BI individuals (*n* = 44, mean = 11.6, *SD* = 3.1) as shown in **Figure 4A**. This observation was confirmed by a main effect of BI (1, 89) = 35.115, *p* < 0.001). There was no main effect of gender (*p* = 0.858) or significant interaction between AMBI scores and gender (*p* = 0.913) on avatar scores. In addition, HA individuals (*n* = 53, mean = 18.1, *SD* = 4.7) also exhibited higher avatar scores than non-HA individuals (*n* = 40, mean = 15.7, *SD* = 5.5) as shown in **Figure 4B**. This observation was confirmed by a main effect of HA (1, 89) = 7.40, *p* < 0.01. There was no main effect of gender (*p* = 0.537) or significant interaction between AMBI scores and gender (*p* = 0.564) on avatar scores.

**FIGURE 5:**
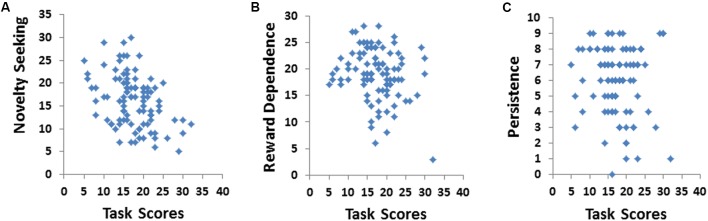
Total scores on the avatar task were not positively correlated with other TPQ subscales. **(A)** Total scores on the avatar task were significantly negatively correlated with novelty seeking (*r* = –0.39). **(B)** Total scores on the avatar task had a non-significant negative correlation with reward dependence (*r* = –0.20). **(C)** Total scores on the avatar task had no significant relationship with persistence (*r* = –0.02).

The TPQ also includes three other subscales: NS, RD, and persistence, which independent of HA. As shown in **Figure 5**, the avatar score was strongly negatively correlated with the NS subscale of the TPQ (*r* = -0.39, *p* < 0.001). A negative relationship between the avatar score and the RD scale fell short of significance (*r* = -0.20, *p* = 0.053). Visual inspection of the of the data revealed an extreme outlier; when the scores for this individual were remove from the analysis, the relationship between the avatar score and the RD scale was further weakened (*r* = -0.15, *p* = 0.134). There was no significant relationship between the avatar score and the persistence scale of the TPQ (*r* = -0.02, *p* = 0.883).

## Discussion

The first goal of this study was to replicate the results of Myers et al. (2016a) concerning the relationship between the avatar task and the AMBI questionnaire with a sample drawn from an undergraduate population at a different geographical location and with different demographic characteristics. Our current sample from a Western university sample was mainly Caucasian and Hispanic, and had virtually equal numbers of males and females with a slightly younger mean age, compared to the mainly African American and Asian, and largely female, sample in Myers et al. (2016a). Additionally, the prior study recruited subjects from a small highly urban campus in the Northeast, whereas subjects in the current study attended a large Western University with both rural and urban components. Despite these differences, the mean AMBI and avatar scores for our current sample were consistent with that reported by Myers et al. (2016a), as were our findings of a strong relationship between BI and avatar scores. This finding thus supports the generalizability of the avatar task to assess behaviorally inhibited temperament in a variety of populations.

Our finding (and that of Myers et al., 2016a) of a strong positive relationship between the avatar scores and the AMBI is perhaps not surprising given that the questions and responses in the avatar task were designed to parallel the AMBI inventory items. Therefore, the second goal of this study was to extend work with the avatar task to examine its relationship to another measure of anxiety vulnerability, namely, HA, as measured by the TPQ. We found that avatar task scores had a strong positive relationship to HA – not as strong as the relationship to BI, but still significant.

In our sample, females exhibited higher levels of HA than males. This finding is similar to the finding of higher BI in females than males in the prior study (Myers et al., 2016a), as well as with the finding of generally higher HA in females reported previously (Nixon and Parsons, 1989; Cloninger et al., 1991). Females also exhibited higher scores on three of the HA subscales: anticipatory worry, fear of uncertainty and fatigability, but not shyness. However, there was no evidence of a gender effect on the avatar task. Females did exhibit a stronger relationship between the avatar scores and the HA scores, compared with males. Analysis of possible gender effects in either under-reporting or over-reporting on the HA and BI measures as compared to the scores on the avatar tasks did not reveal any significant effects for BI, HA or the HA subscales.

We also analyzed the ability of BI and HA to predict avatar scores. While multiple regression analyses did not reveal any significant gains in predictive ability for avatar scores when HA and BI were combined, indirect effects analyses revealed that the HA subscale of fear of uncertainty was a moderate mediating variable in the relationship between AMBI and avatar scores.

This finding is of particular interest given that uncertainty has emerged a common theme in recent findings of an enhancing effect of BI on associative learning in a series of studies involving eyeblink conditioning in humans (Allen et al., 2014, 2016; Holloway et al., 2014). Intolerance of uncertainty has also been proposed as a major component of anxiety disorders (Grupe and Nitschke, 2013). IU has been defined as a tendency to perceive uncertainty as aversive and stressful which results in BI and negative expectations about their possible consequences (Nelson et al., 2015).

In addition to exploring the relationship between HA and BI and avatar scores, we tested the ability of the standard cutoff scores for AMBI and HA to differentiate avatar scores. Our finding that the standard cut-points for BI and HA resulted in groups with significantly different avatar scores is similar the finding of Myers et al. (2016b) that individuals with severe PTSD symptoms exhibited significantly higher avatar scores than those individuals with few or no PTSD symptoms. These findings support the real world applicability of the avatar task as a measure of avoidant behaviors in such a way that correspond to the standard definitions of AMBI and HA based on self-report measures.

There are several limitations to the current study due to possible biases. First, our sample was undergraduate students who were enrolled in introductory psychology courses. These students may lack insight into BI and under-report BI. While not directly addressed in the current study, our current results for BI scores were in the range of those reported by Gladstone and Parker (2005) for a sample with a mean age of 42.7 years. So there does not appear to be a lack of reporting of BI by college age adults as compared to an older sample. In addition, there is some evidence from Myers et al. (2016a) that individuals modulate responding on the avatar task when instructed to respond “as you normally would in real life” as compared to “how you think a typical student at your university would behave.” Subjects’ self-reported BI (based on AMBI) correlated with behavior in the former but not latter case, for both high-BI and low-BI subjects, which would not be expected if subjects were systematically over- or under-estimating their own BI on the questionnaire.

Another possible source of bias in the inventory and avatar data may be social desirability. Social desirability has been reported to be related to low NS scores (Cloninger et al., 1991), which were found to be negatively correlated with avatar scores in the current study. Therefore, it is possible that social desirability would be positively correlated to avatar scores. Social desirability was not studied in the current work, but – as noted above – the prior Myers et al. (2016a) included a condition which asked participants to respond like a hypothetical other (typical college student) instead of as they themselves normally would behave. This manipulation resulted in altered performance in the task which was interpreted as indicating that the instructions to “perform as you normally would” had an effect on responding to the choice points in the avatar task. In addition, these individuals were less likely to report on the post-test questionnaires that the avatar’s behaviors matched their own when responding like a typical college student. Future work could include testing the effects of social desirability on behavior on the avatar task and self-report inventories by measuring social desirability or manipulate typical societal gender norms through text included in the instructions of the avatar game.

Overall, the current study demonstrated a strong positive relationship of behavior on the avatar task with HA, but not with other subscales on the TPQ. The current finding, along with the previous work using the avatar task, demonstrates its effectiveness as a behavioral measure of anxiety vulnerability such as HA (current study), BI (Myers et al., 2016a) as well as PTSD symptoms (Myers et al., 2016b). Completing the computer-based task appears to be less burdensome to participants than completion of the paper and pencil inventories such as the AMBI or TPQ. Prior work has used the AMBI and HA scale of the TPQ to group individuals as anxiety-vulnerable for studies involving eyeblink conditioning (Allen et al., 2014, 2016; Holloway et al., 2014) and computer-based avoidance tasks (Sheynin et al., 2013, 2014a,b). The overall finding from these series of studies is that individuals self-reporting BI or HA exhibit enhanced associative or avoidance learning. The utility of the avatar task as a substitute for paper and pencil inventories such as the AMBI and TPQ should be tested for these experimental protocols with anxiety-vulnerable samples as well as patients with PTSD. The success of the avatar task to extend beyond measures of BI to positively correlate with HA, but not to other personality traits unrelated to anxiety, argues for the further development of computer-based protocols to identify other symptoms related to anxiety disorders. Future work should continue to apply similar computer-based avatar tasks as a viable substitute for paper and pencil inventories which may reduce the effects of avoidant behaviors on self-report measures of anxiety vulnerability.

## Disclosure Statement

The funder had no role in the study design, collection, analysis, or interpretation of the data, in the writing of the report, or the decision to submit the article for publication. The opinions in this article reflect those of the authors and do not necessarily reflect the opinions of the Department of Veterans Affairs or the U. S. Government.

## Ethics Statement

This study was carried out in accordance with the recommendations of the Institutional Review Board of the University of Northern Colorado with written informed consent from all subjects. All subjects gave written informed consent in accordance with the Declaration of Helsinki. The protocol was approved by the Institutional Review Board of the University of Northern Colorado.

## Author Contributions

MA and CM designed the study. MA collected the data. MA, MJ, and CM analyzed the data. MA wrote with original manuscript with a revision by CM and MJ.

## Conflict of Interest Statement

The authors declare that the research was conducted in the absence of any commercial or financial relationships that could be construed as a potential conflict of interest.

## References

[B1] Allen M. T. Miller D. P. (2016). Enhanced eyeblink conditioning in behaviorally inhibited individuals is disrupted by proactive interference following US alone pre-exposures. *Front. Behav. Neurosci.* 10:39 10.3389/fnbeh.2016.00039 PMC4785178 27014001

[B2] Allen M. T. Myers C. E. Servatius R. J. (2014). Avoidance prone individuals self-reporting behavioral inhibition exhibit facilitated acquisition and altered extinction of conditioned eyeblinks with partial reinforcement schedules. *Front. Behav. Neurosci.* 8:347 10.3389/fnbeh.2014.00347 PMC4186341 25339877

[B3] Allen M. T. Myers C. E. Servatius R. J. (2016). Uncertainty of trial timing enhances acquisition of conditioned eyeblinks in anxiety vulnerable individuals. *Behav. Brain Res.* 304 86–91. 10.1016/j.bbr.2016.02.007 26873040

[B4] American Psychiatric Association (2013). *Diagnostic and Statistical Manual of Mental Disorders*, 5th Edn. Washington, DC: American Psychiatric Publication. 10.1176/appi.books.9780890425596

[B5] Barlow D. H. (2002). *Anxiety and its Disorders*, 2nd Edn. New York, NY: Guilford Press.

[B6] Biederman J. Rosenbaum J. F. Bolduc- Murphy E. A. Faraone S. V. Chaloff J. Hirshfeld D. R. (1993). A 3-year follow-up of children with and without behavioral inhibition. *J. Am. Acad. Child Adolesc. Psychiatry* 32 814–821. 10.1097/00004583-199307000-00016 8340303

[B7] Brenner S. L. Beauchaine T. P. Sylvers P. D. (2005). A comparison of psychophysiological and self-report measures of BAS and BIS activation. *Psychophysiology* 42 108–115. 10.1111/j.1469-8986.2005.00261.x 15720586

[B8] Carver C. S. White T. L. (1994). Behavioral inhibition, behavioral activation, and affective responses to impending reward and punishment: the BIS/BAS scales. *J. Pers. Soc. Psychol.* 67 319–333. 10.1037/0022-3514.67.2.319

[B9] Clohessy S. Ehlers A. (1999). PTSD symptoms, response to intrusive memories and coping in ambulance service workers. *Br. J. Clin. Psychol.* 38 251–265. 10.1348/014466599162836 10532147

[B10] Cloninger C. R. (1986). A unified biosocial theory of personality and its role in the development of anxiety states. *Psychiatr. Dev.* 3 167–226. 3809156

[B11] Cloninger C. R. (1987). A systematic method for clinical description and classification of personality variants: a proposal. *Arch. Gen. Psychiatry* 44 573–588. 10.1001/archpsyc.1987.01800180093014 3579504

[B12] Cloninger C. R. Przybeck T. R. Svrakic D. M. (1991). The tridimensional personality questionnaire: U.S. normative data. *Psychiatr. Rep.* 69 1047–1057. 10.2466/pr0.1991.69.3.1047 1784653

[B13] Cloninger C. R. Svrakic D. M. Przybeck T. R. (1993). A psychobiological model of temperament and character. *Arch. Gen. Psychiatry* 50 975–990. 10.1001/archpsyc.1993.01820240059008 8250684

[B14] Degnan K. A. Fox N. A. (2007). Behavioral inhibition and anxiety disorders: multiple levels of a resilience process. *Dev. Psychopathol.* 19 729–746. 10.1017/S0954579407000363 17705900

[B15] Fincham D. Smit J. Carey P. Stein D. J. Seedat S. (2008). The relationship between behavioural inhibition, anxiety disorders, depression and CD4 counts in HIV-positive adults: a cross-sectional controlled study. *AIDS Care* 20 1279–1283. 10.1080/09540120801927025 19012085

[B16] Gladstone G. L. Parker G. (2005). Measuring a behaviorally inhibited temperament style: development and initial validation of new self-report measure. *Psychiatry Res.* 135 133–143. 10.1016/j.psychres.2005.03.005 15922458

[B17] Gladstone G. L. Parker G. B. Mitchell P. B. Wilhelm K. A. Malhi G. S. (2005). Relationship between self-reported childhood behavioral inhibition and lifetime anxiety disorders in a clinical sample. *Depress. Anxiety* 22 103–113. 10.1002/da.20082 16149043

[B18] Grupe D. W. Nitschke J. B. (2013). Uncertainty and anticipation in anxiety: an integrated neurobiological and psychological perspective. *Nat. Rev. Neurosci.* 14 488–501. 10.1038/nrn3524 23783199 PMC4276319

[B19] Hayes A. F. (2013). *Introduction to Mediation, Moderation, and Conditional Process Analysis: A Regression-Based Approach*. New York, NY: Guilford Press.

[B20] Hirshfeld D. R. Rosenbaum J. F. Biederman J. Bolduc E. A. Faraone S. V. Snidman N. (1992). Stable behavioral inhibition and its association with anxiety disorder. *J. Am. Acad. Child Adolesc. Psychiatry* 31 103–111. 10.1097/00004583-199201000-00016 1537760

[B21] Holloway J. Allen M. T. Myers C. E. Servatius R. J. (2014). Behaviorally inhibited individuals demonstrate significantly enhanced conditioned response acquisition under non-optimal learning conditions. *Behav. Brain Res.* 261 49–55. 10.1016/j.bbr.2013.10.041 24275381

[B22] Jorm A. F. Christensen H. Henderson A. S. Jacomb P. A. Korten A. E. Rodgers B. (1998). Using the BIS/BAS scales to measure behavioural inhibition and behavioural activation: factor structure, validity and norms in a large community sample. *Pers. Individ. Dif.* 26 49–58. 10.1016/S0191-8869(98)00143-3

[B23] Kagan J. Reznick J. S. Clarke C. Snidman N. Garcia-Coll C. (1984). Behavioral inhibition to the unfamiliar. *Child Dev.* 55 2212–2225. 10.2307/1129793

[B24] Kagan J. Reznick J. S. Snidman N. (1987). The physiology and psychology of behavioral inhibition in children. *Child Dev.* 58 1459–1473. 10.2307/1130685 3691195

[B25] Karamustafalioglu O. K. Zohar J. Güveli M. Gal G. Bakim B. Fostick L. (2006). Natural course of posttraumatic stress disorder: a 20-month prospective study of Turkish earthquake survivors. *J. Clin. Psychol.* 67 882–889. 10.4088/JCP.v67n0604 16848647

[B26] Kashdan T. B. Morina N. Priebe S. (2009). Post-traumatic stress disorder, social anxiety disorder, and depression in survivors of the Kosovo War: experiential avoidance as a contributor to distress and quality of life. *J. Anxiety Disord.* 23 185–196. 10.1016/j.janxdis.2008.06.006 18676121 PMC2667796

[B27] Leone L. Perugini M. Bagozzi R. P. Pierro A. Mannetti L. (2001). Construct validity and generalizability of the Carver–White behavioural inhibition system/behavioural activation system scales. *Eur. J. Pers.* 15 373–390. 10.1002/per.415

[B28] Maremmani I. Akiskal H. S. Signoretta S. Liguori A. Perugi G. Cloninger R. (2005). The relationship of Kraepelian affective temperaments (as measured by TEMPS-I) to the tridimensional personality questionnaire (TPQ). *J. Affect. Disord.* 85 17–27. 10.1016/S0165-0327(03)00099-5 15780672

[B29] Marshall R. D. Turner J. B. Lewis-Fernandez R. Koenan K. Neria Y. Dohrenwend B. P. (2006). Symptom patterns associated with chronic PTSD in male veterans: new findings from the National Vietnam Veterans Readjustment Study. *J. Nerv. Ment. Dis.* 194 275–278. 10.1097/01.nmd.0000207363.25750.56 16614549

[B30] McCambridge J. deBruin M. Witton J. (2012). The effects of demand characteristics on research participant behaviours in non-laboratory settings: a systematic review. *PLOS ONE* 7:e39116 10.1371/journal.pone.0039116 PMC3378517 22723942

[B31] Morgan B. E. (2006). Behavioral inhibition: a neurobiological perspective. *Curr. Psychol. Rep.* 8 270–278. 10.1007/s11920-006-0062-7 16879790

[B32] Myers C. E. Kostek J. A. Ekeh B. Sanchez R. Ebanks-Williams Y. Krusznis A. (2016a). Watch what I do, not what I say I do: computer-based avatars to assess behavioral inhibition, a vulnerability factor for anxiety disorders. *Comput. Hum. Behav.* 55 804–816. 10.1016/j.chb.2015.07.067 PMC4662559 26622109

[B33] Myers C. E. Radell M. L. Shind C. Ebanks-Williams Y. Beck K. D. Gilbertson M. W. (2016b). Beyond symptom self-report: use of a computer “avatar” to assess post-traumatic stress disorder (PTSD) symptoms. *Stress* 19 593–598. 27594113 10.1080/10253890.2016.1232385 PMC5102780

[B34] Myers C. E. VanMeenen K. M. McAuley J. D. Beck K. D. Pang K. C. H. Servatius R. J. (2012a). Facilitated acquisition of eyeblink conditioning in veterans with high behavioral inhibition, a risk factor for post-traumatic stress disorder (PTSD). *Stress* 15 31–44. 10.3109/10253890.2011.578184 21790343 PMC3364604

[B35] Myers C. E. VanMeenen K. M. Servatius R. J. (2012b). Behavioral inhibition and PTSD symptoms in veterans. *Psychiatry Res.* 196 271–276. 10.1016/j.psychres.2011.11.015 22397911 PMC3361537

[B36] Nelson B. D. Kessel E. M. Jackson F. Hajcak G. (2015). The impact of an unpredictable context and intolerance of uncertainty on the electrocortical response to monetary gains and losses. *Cogn. Affect. Behav. Neurosci.* 16 153–163. 10.3758/s13415-015-0382-3 26438205

[B37] Nixon S. J. Parsons O. A. (1989). Cloninger’s tridimensional theory of personality: construct validity in a sample of college students. *Pers. Individ. Dif.* 10 1261–1267. 10.1016/0191-8869(89)90238-9

[B38] North C. S. Nixon S. J. Shariat S. Mallonee S. McMillen J. C. Spitznagel E. L. (1999). Psychiatric disorders among survivors of the Oklahoma City bombing. *J. Am. Med. Assoc.* 282 755–762. 10.1001/jama.282.8.755 10463711

[B39] O’Donnell M. L. Elliott P. Lau W. Creamer M. (2007). PTSD symptom trajectories: from early to chronic response. *Behav. Res. Ther.* 45 601–606. 10.1016/j.brat.2006.03.015 16712783

[B40] Pérez-Edgar K. Bar-Haim Y. McDermott J. M. Chronis-Tuscano A. Pine D. S. Fox N. A. (2010). Attention biases to threat and behavioral inhibition in early childhood shape adolescent social withdrawal. *Emotion* 10 349–357. 10.1037/a0018486 20515224 PMC3614079

[B41] Reznick J. S. Hegeman I. N. Kaufman E. R. Woods S. W. Jacobs M. (1992). Retrospective and concurrent self-report of behavioural inhibition, and their relation to adult mental health. *Dev. Psychopathol.* 4 301–321. 10.1017/S095457940000016X

[B42] Sheynin J. Beck K. D. Pang K. C. H. Servatius R. J. Shikari S. Ostovich J. (2014a). Behaviourally inhibited temperament and female sex, two vulnerability factors for anxiety disorders, facilitate conditioned avoidance (also) in humans. *Behav. Process.* 103 228–235. 10.1016/j.beproc.2014.01.003 PMC3972301 24412263

[B43] Sheynin J. Beck K. D. Servatius R. J. Myers C. E. (2014b). Acquisition and extinction of human avoidance behavior: attenuating effect of safety signals and associations with anxiety vulnerabilities. *Front. Behav. Neurosci.* 8:323 10.3389/fnbeh.2014.00323 PMC4163982 25309373

[B44] Sheynin J. Shikari S. Gluck M. A. Moustafa A. A. Servatius R. J. Myers C. E. (2013). Enhanced avoidance learning in behaviorally-inhibited young men and women. *Stress* 16 289–299. 10.3109/10253890.2012.744391 23101990 PMC3767128

[B45] Svihra M. Katzman M. A. (2004). Behavioural inhibition: a predictor of anxiety. *Paediatr. Child Health* 9 547–550. 10.1093/pch/9.8.547 19680482 PMC2724161

[B46] Wills T. A. Vaccaro D. McNamara G. (1994). Novelty seeking, risk taking, and related constructs as predictors of adolescent substance use: an application of Cloninger’s theory. *J. Subst. Abuse* 6 1–20. 10.1016/S0899-3289(94)90039-6 8081104

